# Optimizing the quality of clinical studies on oral microbiome: A practical guide for planning, performing, and reporting

**DOI:** 10.1111/prd.12359

**Published:** 2020-11-23

**Authors:** Egija Zaura, Vincent Y. Pappalardo, Mark J. Buijs, Catherine M. C. Volgenant, Bernd W. Brandt

**Affiliations:** ^1^ Department of Preventive Dentistry Academic Centre for Dentistry Amsterdam (ACTA) Vrije Universiteit Amsterdam and University of Amsterdam Amsterdam the Netherlands

**Keywords:** 16S rDNA amplicon sequencing, clinical study design, dental plaque, metadata, microbiome, saliva

## Abstract

With this review, we aim to increase the quality standards for clinical studies with microbiome as an output parameter. We critically address the existing body of evidence for good quality practices in oral microbiome studies based on 16S rRNA gene amplicon sequencing. First, we discuss the usefulness of microbiome profile analyses. Is a microbiome study actually the best approach for answering the research question? This is followed by addressing the criteria for the most appropriate study design, sample size, and the necessary data (study metadata) that should be collected. Next, we evaluate the available evidence for best practices in sample collection, transport, storage, and DNA isolation. Finally, an overview of possible sequencing options (eg, 16S rRNA gene hypervariable regions, sequencing platforms), processing and data interpretation approaches, as well as requirements for meaningful data storage, sharing, and reporting are provided.

## 1 INTRODUCTION

About a decade ago, when the first publications on the oral microbiome using high throughput 16S ribosomal RNA gene amplicon sequencing appeared,^1^, ^2^, ^3^, ^4^ the methodologies of sample processing, sequencing, and the downstream bioinformatic analyses still had to evolve. Today, sequencing costs per megabase have dropped by at least 100‐fold and anyone can send the samples to a sequencing facility and, depending on the services provided, receive fully or partially analyzed data. In the last decade, hundreds of original research articles addressing the oral microbiome have been published, providing an immense volume of information and knowledge on this topic.

However, the problem we are currently facing is that a vast amount of this microbiome data seems to have been created simply because it was both convenient and possible to do so. Frequently this has been done without collecting any oral health‐related information. The data are explored for potential associations and correlations, which are commonly mixed up with causality, leading to overestimation of the clinical relevance and impact of the microbiome on the etiology and pathogenesis of various conditions. Reading such papers raises the question if microbiome sequencing was really necessary. Was this the best methodology for answering the original research question?

With this review, we aim to increase the quality standards for clinical studies with microbiome as an output parameter. To this end, we have critically assessed the current evidence for the best quality practices in oral microbiome studies. This evidence regards the entire process of the clinical study, including the research question, study design, required subject and sample information (metadata), sample type and collection, storage, and processing. We provide a brief overview of the options in the fast‐growing field of sequencing itself and the downstream analyses for novices in the field of 16S rRNA gene amplicon sequencing. Finally, we list open questions that remain to be addressed to further increase the quality of the studies on oral microbiome.

## 2 RESEARCH QUESTION

The first studies on the human microbiome could be categorized as “the phone book” or as “the fishing expedition” studies, as they described the immense and previously unseen bacterial diversity in and on humans. The data from such descriptive studies provided evidence that the attempts of characterizing the human microbiome before the next generation sequencing era were far from complete. New members of the microbiota, never isolated in the laboratory, became “visible” and could be linked to different intra‐oral habitats or pathologic conditions. This has even led to the shift in paradigms on the exclusivity of specific, easily cultivable bacteria in the etiology of oral diseases.

After the first generation of explorative studies, as well as methodological studies focusing on comparing and improving methods in various steps of the fast‐evolving field of microbiomics, we have reached the stage of performing studies driven by research hypotheses. For instance, a recent study very elegantly assessed how a copy‐number variation of the gene encoding for salivary amylase (the AMY1 gene) relates to oral microbiome composition and showed that individuals with a high copy‐number of this gene had a distinct microbiome composition.^5^

After posing the research question, one should investigate the available methodology and carefully judge if obtaining the data on oral microbiome under the circumstances of the planned study will be the best way to provide answers to the question at hand. Perhaps there are other more direct and more economic ways of addressing the same research question. A recent study on the signs of acculturation of Mexican‐American women in the USA^6^ could be used to exemplify this issue. The authors collected detailed acculturation questionnaires regarding language, diet, alcohol consumption, and more. A mouthwash sample, originally collected for genetic analyses, was used for microbiome sequencing. None of the questions in the questionnaires regarded oral health care habits, visits to a dental care professional, or oral complaints. The authors concluded that immigration and adaptation to life in the USA were associated with differences in oral microbial profiles in these women. The more they were accultured, the higher the relative abundance of the genus *Streptococcus* and the lower bacterial diversity they had. However, since streptococci are one of the primary colonizers of teeth, their higher abundance and lower bacterial diversity in general might indicate a higher oral hygiene level, a plausible effect of acculturation. A simple oral hygiene index or at least a question about toothbrushing habits would have been more informative than the wealth of data provided by microbiome analysis.

## 3 CONSIDERATIONS FOR STUDY DESIGN

There are two broad categories of studies for biomedical research, observational and interventional (experimental), each with their own advantages and limitations. In oral microbiome research, the most commonly used designs of observational studies are case‐control studies, cross‐sectional studies, and cohort studies, while interventional studies are usually randomized clinical trials.

### 3.1 Cross‐sectional and case‐control studies

In microbiome studies with a cross‐sectional or case‐control design, two or more groups are compared. These are relatively low‐cost studies generating results quickly, thereby explaining their popularity in the microbiome field. In cross‐sectional studies, the subjects are selected (randomly) from a population, based on a different exposure, for instance, current smokers vs never smokers, measured at one moment in time. The case‐control design involves cases or outcomes of interest, for example, patients with a certain disease, and selected controls. These controls should be comparable (matched) with the cases as much as possible, with the exception of the disease or the condition of interest. A cross‐sectional study is by definition limited to a single measurement of the oral microbiome and cannot assess temporality or causality. The same applies to case‐control studies, but this study design can also be longitudinal. It should be noted that both types are vulnerable to several types of bias.^7^

The high risk of bias in cross‐sectional and case‐control studies is partly related to the complexity and dynamic nature of the host‐microbiome interactions, as well as insufficient matching between the cases and controls. The results of such studies are difficult to reproduce and sometimes results even contradict other studies addressing the same disease or condition. For instance, a recent review identified studies on the oral microbiome of oral squamous cell carcinoma patients in comparison with healthy controls and highlighted large heterogeneity and contradictions in the microbial taxa associated with disease or health among the included studies.^8^ The findings of some of these studies could be biased by poorly matched control groups. Even although the oral squamous cell carcinoma patients and controls in one of the reviewed studies were matched by age and gender,^9^ they were not matched by lifestyle factors known to affect the oral microbiome, namely, tobacco smoking and betel quid chewing (Asian plant compounds with stimulatory substances). The control group consisted of 50% smokers and 28% betel‐chewers, while in the oral squamous cell carcinoma group these were 83% and 90%, respectively.

Another example of poor matching, which biased the study outcomes, ignored the differences in oral health between children with autism and healthy controls.^10^ The authors found significant differences in salivary and plaque microbiome between the two groups, but also reported that children with autism had significantly higher decayed, missing, filled surfaces and gingival bleeding than the controls. The observed differences in microbiome could merely reflect the differences in oral health, and may have nothing to do with autism.

Besides various lifestyle factors and differences in oral health status, numerous other factors are known to affect the oral microbiome. These are summarized in the section on subject and sample metadata (section 3.5) and in Table 1. Because these can potentially influence study outcomes, they should not be ignored when selecting study subjects.

**TABLE 1:** Sample metadata proposed for recording in oral microbiome studies

Metadata for samples in oral microbiome studies
**Date,** time of day, **study aims, inclusion and exclusion criteria for study population, instructions before collection (eg, duration for abstaining from toothbrushing, food or drink intake, chewing gum and mouthwash use), methods of sample collection, processing**
Demographic factors:
‐ **Age** 27, 28
‐ **Gender** 32, 196
‐ Socioeconomic status^29^, ^32^
‐ Education level^32^
‐ Ethnicity^32^, ^36^, ^37^, ^197^, ^198^
General health factors:
‐ **Recent history of exposure to antibiotics** ^39^
‐ **Medication use** ^41^
‐ **Systemic diseases** 72, 199, 200
‐ **Pregnancy** ^44^
‐ Menstrual cycle^196^
‐ BMI^28^
‐ Birth mode^39^
Oral health factors:
‐ **Toothbrushing habits**, interdental cleaning habits **(frequency, time since last brushing)** ^32^, ^100^
‐ **Tongue brushing** ^61^
‐ Type of toothpaste^57^
‐ **Presence** and number **of own teeth, dentures**, dental implants^28^, ^49^, ^51^
‐ **Bleeding gums while brushing**, GI (BOP)^15^, ^47^
‐ **Presence of tooth decay**, caries indices (DMFS, ICDAS)^11^, ^28^, ^104^, ^201^, ^202^
‐ **Oral hygiene level** (plaque index)^54^
‐ **Diagnosis of periodontal disease**, periodontal pocket status (PPD, BOP, CAL)^47^
‐ **Dry mouth complaints**, salivary flow rate^52^
‐ **Tongue** and lip **piercings** ^62^
‐ **Fixed orthodontic appliances** 64, 100
‐ **Presence of intra‐oral lesions** ^203^
Lifestyle factors:
‐ **Cigarette smoking, smokeless tobacco use** 68, 69, 71, 72, 73, 74, 75, 78, 204, 205
‐ Alcohol consumption^80^, ^206^
‐ Betel or khat chewing^81^, ^83^
‐ Diet, **sugar intake frequency**, time since last food intake, breastfeeding^32^, ^84^, ^85^, ^87^, ^88^, ^89^, ^91^, ^92^, ^93^
Other factors:
‐ Climate, season, time of day^97^, ^98^
‐ Tap water quality and composition^100^

Abbreviations: BMI, body mass index; BOP, bleeding on probing; CAL, clinical attachment loss; DMFS, decayed, missing, filled surfaces; ICDAS, International Caries Detection and Assessment System; PPD, periodontal probing depth; GI, gingival inflammation; ICDAS, International Caries Detection and Assessment System; PPD, periodontal probing depth.

In **bold** – the minimum information that should be recorded and reported for reliable data interpretation.

### 3.2 Cohort studies

Cohort studies are considered the gold standard for observational research and can be performed both retrospectively and prospectively. In retrospective cohort studies, the oral samples, stored in a biobank, have usually been collected for purposes other than oral microbiome analyses, for instance, for genetic assessment as in the acculturation study mentioned above.^6^ These studies frequently did not collect any information regarding the oral health of individuals, as the purpose for storing the sample in the biobank was related to general health.

Prospective cohort studies follow a specific outcome that has been planned upfront and the study subjects are examined and their microbiomes assessed as they get older, at several time points. Consequently, prospective cohort studies usually create high quality data accompanied by the appropriate metadata, allowing both assessment of the role of the oral microbiome in etiology of the disease and potential for disease risk prediction. For instance, a recent publication on an Australian cohort of 134 children was followed from 2 months until 4 years of age demonstrated the potential of salivary microbiome in predicting the development of early childhood caries.^11^

### 3.3 Interventional studies

Interventional or experimental studies aim to assess the therapeutic or preventive effects of specific interventions by the investigator. The most common and strongest interventional study design is a randomized controlled trial, which is preferably triple blinded, for study subjects, clinical investigators, and (bio)statisticians. A traditional randomized controlled trial involves study subjects randomly allocated into two or more groups, where the intervention (the test) is compared with a control (positive or negative), or to no intervention at all. The strengths of this design are allocation concealment, the possibility to measure compliance and dropout, to analyze results by intention to treat, and to assess each treatment arm in the same manner, preferably using good clinical practice guidelines. A crossover randomized controlled trial design is a variant of a randomized controlled trial where the same individual is allocated randomly to start with one intervention, followed by a sufficiently long washout period, and completing with the other intervention. There are several aspects to consider when planning an intervention study.

#### 3.3.1 The observer effect

In oral health research, the study subjects may change their usual behavior, for instance, by temporarily improving their oral hygiene practices just because they are being meticulously observed by dental professionals. This is evidenced by an improved oral health status, such as lowered gingival bleeding and plaque scores in the first week of the study on healthy Dutch young adults (the authors' unpublished findings). As the study proceeds, the clinical outcomes (eg, bleeding, plaque indices) tend to increase, suggesting that the individuals get used to being observed and their behavior returns to what was normal for them. Because the clinical changes are related to changes in the microbial composition of dental plaque,^12^ this implies that the composition of the samples collected at the start of the study may differ from those collected later, even without an active intervention. A solution to circumvent this issue could be introducing a “false” start of the study, followed by a “real” start, such as a second baseline visit, once the study subjects adapt to being observed. To date, there is no evidence available for the optimal number of visits or duration of such an adaptation period.

#### 3.3.2 Temporal stability

Although the oral microbiome is shown to be the most stable niche among different niches of the human body,^13^ natural temporal variability can still introduce noise in measurements while assessing the effects of interventions. When the tongue dorsum of 85 adults was sampled weekly for 3 months, the microbiome variability appeared to be highly individualized.^14^ Replicated samples could be collected for the assessment of natural temporal variability of each individual before entering the intervention phase. This enables assessment of the normal variability and the changes in microbial composition introduced by the intervention. Each individual becomes their own control in the measurement of the effect of the intervention, which allows a reduction in sample size (see the section 3.4.1).

#### 3.3.3 Population normalization at the start of the study

Another issue, relevant for interventional studies, regards oral prophylaxis prior to abstaining from the oral hygiene measures within an experimental gingivitis protocol. Some of these studies perform professional tooth cleaning at the start of the study in order to normalize their population to the same plaque level before entering the nonbrushing phase,^15^, ^16^ while others do not.^17^, ^18^ The magnitude and duration of the changes introduced by the prophylaxis step to the natural oral ecosystem will be highly individual and might introduce additional noise to the microbiome data, potentially leading to an underestimation of the effects of the intervention being studied or to clinically less relevant findings.

#### 3.3.4 Intervention

Additionally, the duration and the dosage of an intervention is frequently either arbitrarily chosen or based on the estimated clinical effects or even preliminary findings from in vitro experiments. However, in cases where subtle ecological changes are expected, for example, as a result of food supplements containing pre‐ or probiotics, the ecosystem might need a substantial time to remodel from one state to another. This would mean that longer intervention periods are required before any changes in composition can be measured. Therefore, when assessing novel ecological interventions, researchers might prefer to evaluate the minimum exposure required in a pilot experiment before setting up a much more costly and elaborate full‐scale randomized controlled trial.^19^ The data obtained from such a pilot would also provide the basic information needed for power estimation of the main study.

### 3.4 Sample size determination

Running a clinical study, especially a randomized controlled trial, is one of the most expensive forms of biomedical research. Besides the high costs involved, clinical research can only be performed if the rights, safety, and well‐being of the research subjects are protected in agreement with the declaration of Helsinki.^20^ From the perspective of costs, as well as from the point of ethics, studies which are too large are undesirable. However, by including too few subjects, the effects of the intervention or the differences between the cases and controls may be missed, biasing the conclusions of the study. Proper sample size determination before planning the study would avoid these issues. However, assessment of the power in studies with microbiome as the main output parameter is not straightforward. Difficulty lies in the high inter‐individual variance and multidimensionality of the data. Each individual has a slightly different set of taxa (eg, operational taxonomic units), resulting in a data set (eg, an operational taxonomic unit table) with a high number of zero counts per operational taxonomic unit (high sparsity of data) within an individual sample. Additionally, functional redundancy (different taxa performing similar biologic functions within an ecological niche) among the members of the oral microbial community will lead to different taxa in different individuals that respond to the same intervention.

#### 3.4.1 Power analysis tools for microbiome studies

To date, reporting of power analyses in the methods sections of studies with microbiome as the main output parameter is scarce and is mainly limited to methodology papers, describing various tools for sample size calculations using microbiome data.^21^, ^22^, ^23^, ^24^ Some of these tools use frequency distributions of individual taxa, for example, operational taxonomic units, for sample size calculation.^21^, ^24^ Others are based on the measurement of the change in the community structure, for example, on pairwise distances between samples instead of changes in the relative abundances of individual taxa.^21^, ^22^, ^23^ A tool developed by Mattiello et al^21^ allows stratification of samples into subgroups, for example, by gender, age, or any other parameter, thus increasing the statistical power considerably. To avoid excessive false‐positive findings that could arise because of a poorly chosen pairwise distance method, another distance‐based power analysis tool—PERMANOVA‐S—was developed that allows combination of multiple distances from various distance‐based methods.^22^

An alternative solution for estimating an effect size for microbiome studies involves subsampling the data,^25^ as in the Evident software package (https://github.com/biocore/Evident), which uses Monte Carlo simulations to estimate the variance and to explore both sampling depth and the number of samples needed for visual separation between groups.

A recent study addressed the effects of intrinsic temporal variation on sample size in longitudinal microbiome studies.^26^ The authors estimated intraclass correlation coefficients between various metrics such as most abundant taxa, alpha (within‐sample), and beta (between‐samples) diversity from microbiome data from different studies. To calculate intraclass correlation coefficients, temporal replicates and also preferably technical replicates are required. The authors showed that a higher intraclass correlation coefficient implies larger statistical power and smaller bias in estimating the effect of an intervention.

While the list of power tools based on microbiome data is growing and several user‐friendly web‐based interfaces have become available, there is, however, no consensus yet on the optimal methodology for sample size determination for oral microbiome studies.

### 3.5 Subject and sample metadata

**FIGURE 1:**
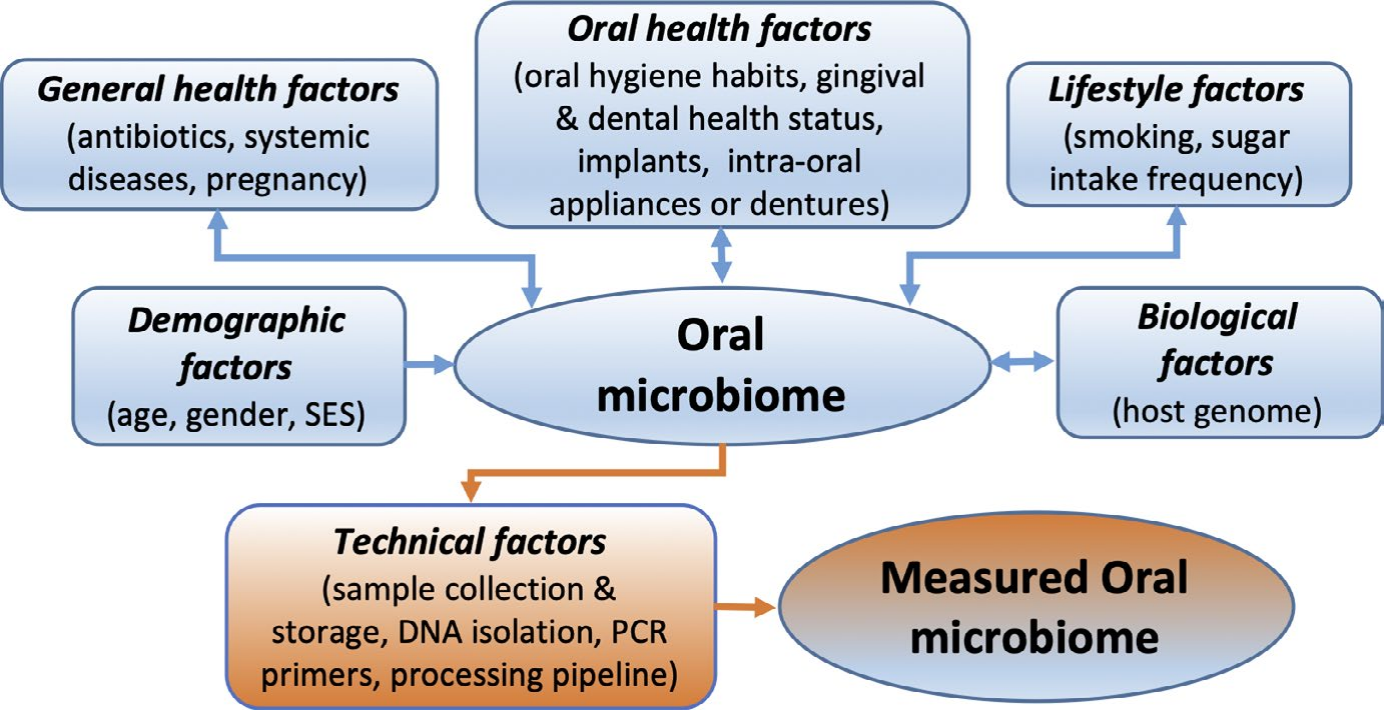
Schematic representation of the main factors that are known to influence the oral microbiome. In blue, factors associated with the individual and, in orange, technical factors introduced during the study. SES, socioeconomic status. A detailed list of subject‐related factors is presented in Table 1

The data created by sequencing of any microbial communities, including those of oral origin, remain just plain data without scientifically reliable interpretation if they lack accompanying information—the metadata—of the samples and study population (Table 1). In this section, we provide a summary of the current evidence for the measurable effects of demographic factors, general and oral health, as well as behavior of the individual on the oral microbiome (Figure 1). Based on this evidence, we have listed the optimal and the minimum required information (Table 1, minimum information in bold) that should be recorded when performing a clinical study with oral microbiome as one of the study outcomes.

#### 3.5.1 Basic information

On all occasions, basic information about the study should be provided. This includes the sample collection date, a description of the study aim(s), inclusion and exclusion criteria of the study population, and the instructions given to the study subjects before the sample collection, such as duration of abstaining from toothbrushing, food or drink intake, chewing gum and mouthwash use.

#### 3.5.2 Demographic factors that have been shown to affect oral microbiome

##### Age

Age is one of the standard parameters that is always recorded. This, of course, is highly relevant information, especially in case‐control studies where groups should be matched by age. The oral microbiome has been shown to vary according to the age of the study subjects, especially when infants, children at various dentition stages (deciduous, mixed, or permanent dentition), adolescents, and younger and older adults are compared.^27^ A large population‐based study on 2343 Japanese adults aged 40 years and older showed that even in this relatively homogeneous group salivary microbiome differed significantly by age.^28^

##### Gender

Most of the studies either do not assess if there is a relation between the gender and oral microbiome composition, or do not find any considerable differences.^29^, ^30^ A recent study on 268 young, orally and systemically healthy Dutch adults found no difference in alpha diversity in unstimulated saliva by gender, but did identify 65 operational taxonomic units that differentiated between males and females.^31^ Another recent study, investigating oral microbiome of 282 US subjects, confirmed that there were no differences in alpha diversity, while some taxa differentiated males from females.^32^

##### Socioeconomic status and education level

A demographic factor known to be indirectly related to general and oral health involves both the socioeconomic status and education level of the individual.^33^, ^34^, ^35^ Several studies report a relation between income or education level and oral microbial composition: a microarray study on stimulated saliva of 292 Danish adults with low levels of caries and periodontitis found that socioeconomic status explained 20% of the overall variance in salivary microbiome.^29^ A more recent study, using amplicon sequencing, also found differences in oral microbiome related to income and education level of the study subjects.^32^

##### Race or ethnicity

Race or ethnicity of the individual is a demographic factor with relatively strong evidence for the relationship with oral microbial composition. The Human Microbiome Project found differentially abundant taxa at every habitat of the body, including different oral niches, when comparing non‐Hispanic white (Caucasian), non‐Hispanic black (African American), Asian, Mexican, and Puerto Rican ethnicities.^30^ A study on supragingival and subgingival plaque and saliva from 192 subjects belonging to four major ethnicities in the USA also found taxonomic differences by ethnicity.^36^ Interestingly, even genetically closely related populations, such as Japanese and South Korean orally healthy adults, differ in their salivary microbiome.^37^

#### 3.5.3 General health factors known to affect oral health and oral microbiome

Any clinical study should have clearly predefined inclusion and exclusion criteria regarding general health of the study subjects, because of a strong relation between the oral and general health of the individual.^38^ Previous exposure to antibiotics^39^ and current medication use should be recorded. The effects of medication have been shown on the fecal microbiome,^40^ while reports of medication use on the oral microbiome are still scarce. A recent interventional study on oral microbiome found that daily use of esomeprazole, a proton pump inhibitor, for 4 weeks, led to an increase in *Streptococcus* and a decrease in *Veillonella* and *Neisseria*, while the bacterial diversity was significantly reduced.^41^

##### Exposure to antibiotics

Regarding antibiotic exposure, there is no consensus on the minimum duration between exposure and enrollment in studies on oral microbiome. In studies on the gut microbiome, an arbitrary period of 6 months since the antibiotic exposure is frequently used, although shorter periods have also been applied.^19^ In a randomized controlled trial, healthy individuals were exposed to either clindamycin, ciprofloxacin, amoxicillin, minocycline, or placebo, and their fecal and salivary microbiota were assessed during 1 year.^42^ Antibiotics had very limited impact on the salivary microbiome, with effects on bacterial diversity and community structure being measurable right after the exposure, but already becoming undiscernible after 1 month, while the gut microbiota needed up to 1 year to recover from the exposure to some of the antibiotics. The response to antibiotics is highly individual and might also be influenced by the underlying infection when antibiotics are clinically prescribed instead of being tested on healthy individuals. Currently, most studies on the oral microbiome use a minimum of 2 or 3 months since the end of the antibiotic therapy as inclusion criterium.

##### Systemic diseases

A history of systemic diseases, such as diabetes, rheumatoid arthritis, and cardiovascular disease, that have implications for oral health,^38^ should be recorded. A general characteristic of well‐being such as frailty has also been shown to relate to differences in oral microbiome.^43^ The oral microbiome was shown to differ by the body mass index of the study subjects.^28^ Pregnancy is a typical exclusion criterion, as changes induced by pregnancy hormones on the entire body, including the oral microbial ecosystem,^44^ will seriously bias the study outcomes. Additionally, if the study population involves infants and young children, information regarding the mode of birth^39^, ^45^, ^46^ and predelivery antibiotic prophylaxis as a result of a Cesarian section might be relevant to record.

#### 3.5.4 Oral health‐related factors known to affect oral microbiome

##### Oral health status

One of the most important factors with the strongest evidence for an impact on the oral ecosystem and its microbiome is the oral health status of the individual. Gingival and periodontal health,^15^, ^47^ dental caries,^28^, ^48^ presence of intra‐oral implants^49^ and the health status of the peri‐implant sulcus,^50^ number of teeth present, edentulism, and dentures,^28^, ^51^ have all been shown to have a strong impact on the oral microbiome. Another obvious factor influencing the oral ecosystem is the salivary flow rate. To date, evidence for its effect on oral microbiome is inconclusive. One study reported that salivary flow rate does indeed affect salivary microbiome,^52^ while another study failed to find any differences.^53^

##### Oral hygiene habits

Daily oral hygiene practices (eg, frequency of toothbrushing, efficacy of plaque removal) affect the oral health of the individual. Therefore, it is not surprising that the salivary microbiome has been shown to reflect the oral hygiene level of the individual, both in children and in adults.^28^, ^54^ Nevertheless, numerous published studies fail to record metadata on oral health or oral hygiene behavior of the subjects. For instance, no oral health‐related information was collected in a recent study comparing the salivary microbial composition of chronic fatigue syndrome patients with that of age‐, gender‐, and body mass index‐matched healthy controls.^55^ Because chronic fatigue syndrome is a neurological disorder, differences in the attitude and level of self‐performed oral care could be the most likely explanation for the observed differences in salivary microbiome between the cases and controls, and should have been assessed at least additionally to the microbiome analyses. Similarly, the oral microbiome from mouthrinse samples of diabetics was compared with those of obese and nonobese nondiabetics without any oral status assessment.^56^

Prolonged use of a particular oral health care product with additives aiming at ecological modification of the oral ecosystem has also been shown to affect oral microbial composition. For example, use of a toothpaste containing enzymes and proteins^57^ or a toothpaste containing arginine has been shown to affect supragingival plaque composition,^58^, ^59^ while twice‐daily mouthrinse with amine fluoride and stannous fluoride for 2 weeks resulted in microbial shifts in tongue and saliva.^60^

Frequency of tongue brushing was recently shown as not only able to affect tongue microbiome composition, but was also related to the effects of chlorhexidine mouthwash on the microbial composition.^61^ Besides recording tongue cleaning habits, the presence of tongue piercings should also be noted and preferably used as exclusion criterion, as these could be potential reservoirs of taxa associated with periodontitis.^62^ In addition to piercings, wearing appliances such as fixed braces during orthodontic treatment should be considered as exclusion criterion, as these increase plaque retention and affect the oral microbial ecosystem.^63^, ^64^

##### Self‐reported oral health status

There is no doubt that oral health and oral hygiene behavior have a direct impact on the oral microbiome and vice versa. However, it is not always possible to conduct a clinical intra‐oral examination. In such cases, at least the minimum information on oral care habits and oral health status should be obtained through questionnaires that have been validated for self‐assessment of oral health, for example, for the periodontal status^65^, ^66^ and dental caries experience.^67^ To date, we are not aware of a single validated questionnaire that could be used for all oral health‐related factors, as shown in Table 1, and therefore a composite of questions from different questionnaires should be used.

#### 3.5.5 Lifestyle factors that have been shown to affect oral microbiome

##### Smoking

Currently, there is ample evidence that smoking tobacco not only has devastating effects on general health, but also on the oral health and oral microbiome.^68^, ^69^, ^70^, ^71^, ^72^, ^73^, ^74^, ^75^, ^76^ Because smoking cessation also leads to measurable changes in oral microbiota,^77^ it should be noted how long ago it was since an individual stopped smoking.

Modern alternatives to tobacco smoking, such as electronic cigarette smoking, have not yet been investigated in any great detail, although a pilot study on this topic did not find any difference between electronic cigarette smokers (N = 10) and nonsmoking controls (N = 10).^69^ Besides conventional tobacco cigarette smoking, smoking of dokha (an Arabic tobacco product) was also shown to affect oral microbiome and lead to dysbiosis, while microbiota of shisha (a water pipe) smokers did not differ from nonsmokers in a study with 330 subjects from the United Arab Emirates.^78^

##### Alcohol consumption

Another lifestyle factor with prominent health effects is alcohol consumption. In the oral cavity, alcohol is metabolized by oral bacteria into acetaldehyde, which is a known carcinogen.^79^ A study of 1044 US adults found that heavy and moderate drinkers had higher alpha diversity and that their oral microbiome differed from nondrinkers.^80^ It should be noted, though, that this study lacked any information regarding the oral health status or oral care habits of the individuals and therefore the differences observed could have been biased by these factors. To correct for these confounders the authors used surrogate oral health indicators: presence of *Porphyromonas gingivalis* and *Aggregatibacter actinomycetemcomitans* for periodontal disease and a high proportion of *Streptococcus mutans* for caries, which is a very simplified view of oral diseases.

##### Chewing psycho‐stimulatory substances

In some cultures, but especially in south Asia, southeastern Asia, and the Pacific, areca nut or betel quid chewing is a widespread habit, and has become a leading cause of oral cancer in those areas of the world.^81^ Differences in the oral microbiome of betel chewers compared with the control group were reported.^81^ Chewing of leaves and twigs of khat that provide amphetamine‐like effects is another habit gaining popularity among certain cultures^82^ and is shown to affect the oral microbiome.^83^

##### Diet

Diet has been shown to explain a considerable part of the variation in gut microbiome composition.^40^ The strongest evidence of the effects of dietary components on the oral microbiome regard sugar intake.^84^, ^85^, ^86^ Differences have been found among the oral microbiomes obtained in the Philippines from hunter‐gatherers, who rely on fishing, hunting, and gathering, compared with traditional farmers who rely on cultivated rice and vegetables in their diet, and those living on a Western diet.^87^ Differences in self‐reported bovine milk intake were associated with oral microbial differences in Swedish adolescents.^88^ A study following African celiac children, who switched from an African‐style, gluten‐free diet, known to contain noncertified foods contaminated with gluten, to an Italian‐style diet of certified gluten‐free products for 60 days, reported changes in their salivary microbiome and metabolome composition.^89^

However, a study of Italian subjects following a habitual omnivore (N = 55), ovo‐lacto‐vegetarian (N = 55), or vegan (N = 51) diet for at least 1 year before the sample collection found no differences in their salivary microbiome.^90^ By contrast, a more recent study comparing healthy Danish vegans (N = 78) and omnivores (N = 82) did find differences in their salivary microbiomes.^91^ Furthermore, a study of 282 US subjects assessing the effects of the frequency of consumption of beverages containing sugar, meat, poultry, fish, vegetables, and fruits in the week prior to the sample collection found taxa that differentiated the dietary habits.^32^ Analysis of food frequency questionnaire data in comparison with oral microbiome found that saturated fatty acid and vitamin C intake correlated with differences in microbial composition in a study of 182 Americans.^92^

Breastfeeding has been shown to lead to different oral microbiota compared with formula‐fed infants.^93^ A recent study found that effects of partial vs no breastfeeding were still evidenced in the salivary microbiomes of 2‐ and 7‐year‐olds.^39^

A recent randomized controlled trial with Estonian schoolchildren assessed the long‐term effects of candies containing different polyols—erythritol, xylitol, or sorbitol—on salivary microbiome composition.^94^ The group consuming erythritol‐containing candies for 3 years during school days had the microbiome deviating the most from the rest and the lowest caries scores at the end of the intervention.

#### 3.5.6 Other factors potentially influencing oral microbiome

##### Climate, season of enrollment, and time of the day

It has been reported that populations living in different geographic and climatic environments (Alaska, Germany, or Africa) differ in their salivary microbiomes.^95^ This study, however, did not account for any oral health status‐related confounders.

The composition of some human microbial habitats has been shown to depend on the season of enrollment into the study, for example, in the case of the nasal microbiome of infants.^96^ Currently, there is not enough evidence to conclude that the oral microbiome is similarly affected. However, higher bacterial DNA concentration was measured in the saliva of study subjects taken in February compared with other periods of the year during a sample collection occurring every 2 months for 1 year.^97^ This could be attributed to seasonal differences in immune fitness.

To date, one study has found effects of the time of the day on oral microbiome,^98^ while another study failed to replicate this.^99^

##### Tap water quality and composition

An interesting observation arose from a citizen science project in Spain involving 1555 adolescents (aged 13‐15 years) and their teachers from 40 Spanish schools.^100^ Their salivary microbiome varied not only by different lifestyle and oral hygiene habits, but also by certain parameters (eg, alkalinity, water hardness) of the tap water in the municipality they lived in. The authors concluded that drinking water may contribute to the shaping of the oral microbiota.

### 3.6 Sample type choice

#### 3.6.1 Intra‐oral niches

**FIGURE 2:**
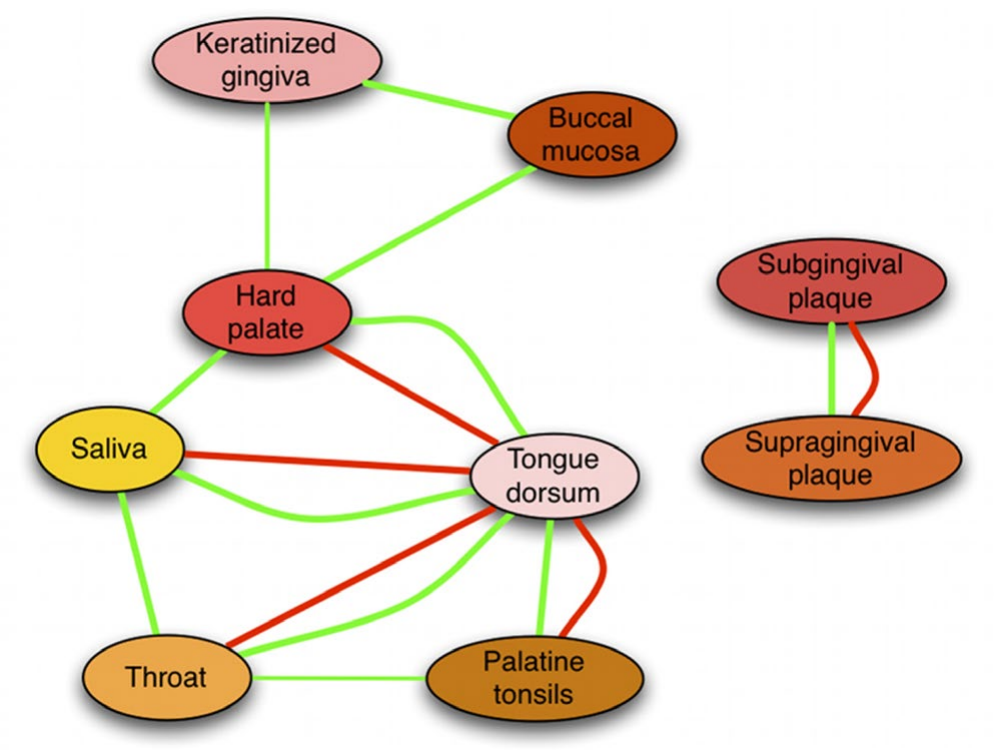
Significant cross‐niche correlations among bacterial composition in nine different oro‐pharyngeal sample types collected within the Human Microbiome Project. Green: significant co‐occurrence; red: co‐exclusion of different bacterial taxa (part of Figure 5 from Faust et al)

The oral cavity is a complex ecosystem, consisting of different niches with compositionally different communities, where shedding (mucosal tissue) and nonshedding (dental hard tissue) surfaces form two major, compositionally distinct niches (Figure 2).^101^ Thus, a universal “oral microbiome” sample that would represent the entire ecosystem does not exist. Besides these tissue‐related differences, there is a spatial gradient, shaped by salivary flow, from the front to the back of the mouth.^102^ The anatomic location (eg, upper buccal molar surface vs lower lingual) has been shown to affect the composition of supragingival plaque within the same individual.^103^

To date, numerous types of samples have been used to study the oral microbiome, each with their own advantages and limitations (Table 2). Often, based on the research question and hypothesis, it is straightforward regarding which sample type to collect. For example, to characterize biofilms on early and dentinal caries lesions, the best choice is to collect a site‐specific plaque sample from the surface of a white spot lesion and a dentin caries lesion, respectively.^104^ Frequently, though, there are practical limitations related to the study setup, for example, home sampling by subjects, which would be decisive regarding which samples (not) to collect.

**TABLE 2:** Advantages and disadvantages of major sample types used in oral microbiome studies with an example reference per sample type

Sample	Advantages	Disadvantages
Unstimulated saliva^106^	A proxy for oral microbiome; noninvasive; self (home) sampling and repeated sampling possible	Does not represent a specific niche; relatively time‐consuming and drooling might feel uncomfortable (5 min)
Stimulated saliva^106^	A proxy for oral microbiome; noninvasive; self (home) sampling and repeated sampling possible; faster collection and less discomfort than unstimulated saliva	Does not represent a specific niche; requires a gum base or parafilm; more diluted than unstimulated sample; chewing activity might affect sample content
Oral rinse/mouthwash^108^	A proxy for oral microbiome; noninvasive; self (home) sampling and repeated sampling possible; fast (30‐60 s)	Does not represent a specific niche; requires a mouth rinse, constituents of which might affect the composition; more diluted than saliva sample
Pooled supragingival plaque^118^	Represents an intra‐oral niche, relevant for oral health; contains low human DNA proportion	Sample composition depends on time since toothbrushing and brushing efficiency; self‐sampling possible but less reliable than by trained researcher; sampling all surfaces time‐consuming; repeated sampling possible only after regrowth of dental plaque
Site‐specific supragingival dental plaque^104^	Represents a specific dental site; allows discrimination between caries lesions and intact surfaces	Sampling requires a trained researcher and a clinical setting; surfaces need to be cleaned and diagnosed in a different appointment; time since cleaning needs to be standardized; low sample biomass; repeated sampling possible only after regrowth of dental plaque
Subgingival plaque^13^	Represents an intra‐oral niche, relevant for oral health; possible to sample specific sites repeatedly if using paperpoints	Sampling requires removal of supragingival plaque by a trained researcher in a clinical setting; low sample biomass in cases without periodontal pockets; use of paperpoints – risk of DNA contaminants; use of curettes – risk of damage to periodontium and not suitable for frequent resampling
Interproximal plaque^207^	Represents an intra‐oral niche, relevant for oral health; possible to sample specific interdental area and assess effectiveness of anti‐biofilm measures on plaque stagnation sites; high bacterial diversity	Interdental hygiene habits affect sample composition; not possible to sample with deficient restorations; low sample biomass; highly trained researcher required; repeated sampling limited – requires accumulation of mature biofilm; for replicates within 1 wk – comparable but different sites should be sampled
Tongue swab^13^	Represents an intra‐oral niche; easy to sample; self‐sampling and repeated sampling possible; most stable intra‐oral niche in general; sufficient material to sample	Tongue brushing habit affects the composition; low similarity with dental plaque composition; high compositional stability might limit the applicability in intervention studies; high human DNA proportion
Buccal swab^13^	Represents an intra‐oral niche; relatively easy to sample; self‐sampling and repeated sampling possible	Low bacterial diversity; potential contamination with other surfaces (eg, teeth) and saliva will affect composition; high human DNA proportion
Tonsillar swab^13^	Represents an intra‐oral niche, relevant for oral and general health; microbial community not disturbed by toothbrushing	Requires trained researcher to sample; uncomfortable and uneasy sample collection procedure; repeated sampling only possible after considerable time
Palatal swab^13^	Represents an oral niche, specifically relevant for oral health of full upper denture wearers; relatively easy to sample; repeated sampling possible	Low bacterial diversity; contamination with other surfaces (eg, tongue) possible; contains high human DNA proportion
Dental calculus^123^	Represents calcified dental supra‐ or subgingival biofilm; allows comparisons with ancient calculus in molecular anthropology and archeology studies	Sample collection requires removal of plaque and calculus by trained researcher in a clinical setting; repeated sampling from the same site not possible; requires extensive sample processing compared to other samples
Denture surface swab^51^	Represents a niche in (partially) edentate individuals; relevant for oral and general health; relatively easy to sample	Denture hygiene affects sample composition; standardization of sample collection procedure required
Mucosal citobrush^208^	Captures host‐microbe interactions; relatively easy to sample; allows sampling a specific mucosal area	Low bacterial diversity; low bacterial and high human DNA proportion
Sub‐mucosal biopsy^209^	Captures host‐microbe interactions; targets specific mucosal area of interest	Invasive method; disrupts mucosal tissue; contains low bacterial and high human DNA proportion

#### 3.6.2 Saliva and oral rinse samples

Two of the sample types especially gaining interest in large cohort studies are saliva and its surrogate, an oral rinse. Both are relatively easy to collect, also by the study subjects themselves. For saliva, all‐in‐one saliva collection and DNA stabilization kits are commercially available, allowing shipment of the samples in ambient temperature, such as OMNIgene Oral OM‐501 kit for unstimulated saliva collection (DNA Genotek, Ottawa, ON, Canada). Both methods are discussed below.

##### Stimulated and unstimulated saliva

It should be realized that saliva itself is not a niche. It is a continuously produced bodily fluid, with microbial and host cells that are dislodged from the oral surfaces and collected together with salivary components. Both its volume and biochemical composition will be different if salivary secretion is passive or stimulated, for example, by a masticatory or gustatory stimulus.^105^ To date, reports regarding the effects of activated secretion by chewing on microbial composition are contradictory: Belstrøm et al^106^ compared paraffin chewing‐stimulated saliva with unstimulated saliva, collected by passive drooling in 20 healthy adults, and found no difference between the two types of samples. By contrast, Gomar‐Vercher et al^107^ reported highly significant differences between the two types of samples collected from 10 12‐year‐old children. These differences might be a result of the way the unstimulated sample was collected in the latter study: instead of a passive drooling for 5 minutes, the samples were collected by depositing three sterile paperpoints for 30 seconds on the floor of the mouth.

##### Oral rinse sample

In some cases, however, saliva collection is either too time‐consuming or the study subjects suffer from painful mucosal lesions or dry mouth, precluding conventional saliva sample collection. An alternative way of collecting salivary microbes is to use an oral rinse with a predetermined volume of fluid.^108^

Oral rinse samples have been collected in several large cohort studies, originally to collect human genetic material and not to analyze the salivary microbiome. For instance, in 50 000 subjects from a cohort initiated in 1992 by the American Cancer Society, human cells were collected using an oral rinse with a commercially available mouthwash, Scope (Procter & Gamble), which contains 15 wt% alcohol and cetylpiridine chloride.^109^ The antimicrobial activity of the mouthwash allowed home‐sampling and shipment of the samples in ambient temperature without damaging the sample material. Decades later, Fan et al^110^ assessed the applicability of the samples for oral microbiome analyses by comparing unstimulated saliva with the mouthwash sample, obtained by vigorous swishing with 10 mL Scope for 30 seconds from 10 individuals. The authors found no difference between the two sample types and concluded that the frozen mouthwash samples are suitable for oral bacterial microbiome analysis.

Another recent study, though, comparing Scope mouthwash samples with unstimulated saliva, did report differences in microbial composition between these two sample types.^111^ Yu et al^112^ compared Scope mouthwash samples with saliva and seven other intra‐oral niches collected according to Human Microbiome Project sample collection protocol in 41 healthy individuals. The authors concluded that the oral rinse sample had a higher alpha diversity than the other samples and mainly resembled the saliva sample, followed by the hard palate and buccal mucosa samples.

Another study compared saliva collected by passive drooling, active spitting, and 10 mL saline rinse for 1 minute.^113^ Higher amounts of total and bacterial DNA were obtained from the oral rinse samples, followed by spit and drool samples. The alpha diversity tended to be higher in the oral rinse samples than the others, while microbial composition was driven by individual subject and did not differ by sample type.

In summary, unstimulated saliva and the sample collected by oral rinse seem to differ, especially in the DNA yield and bacterial diversity, but compositional differences are minor.

#### 3.6.3 Niche‐specific oral samples

As stated above, saliva or oral rinse samples do not represent a certain intra‐oral niche, but compositionally they do resemble samples from the mucosal surfaces.^101^, ^112^ Oral diseases involve specific surfaces and are often biofilm‐initiated, therefore site‐specific samples are frequently preferred to salivary or rinse samples (Table 2). Some studies using saliva have failed to discriminate differences that were clinically discernable, for example, in the cases with and without caries, and conclude that saliva is not the best sample for that purpose.^114^ Again, depending on the aims and hypotheses of the study, the most appropriate sample type(s) should be selected. If the aim is to assess overall microbiome diversity, then collecting multiple samples from different niches will be the most appropriate approach. One specific niche or sample type might be more discriminative than another when comparisons of healthy vs diseased populations are performed or following the effects of an intervention on the oral microbiome. To facilitate the selection of such a niche, the comparison of multiple niches has been performed by several studies, discussed below, both in health and disease, in adults and children.

##### Niche‐specificity in children

The oral microbiome in children undergoes various developmental stages, following the anatomic changes occurring because of teeth eruption and growth, and changes in feeding habits.^115^ Older studies using the DNA‐DNA checkerboard technique have addressed issues of niche‐specificity in this population. For instance, unstimulated saliva, supragingival and subgingival plaque, tongue dorsum, and a mucosal swab over cheeks, lips, and palate were collected from 93 children aged 3‐12 years and assessed using DNA‐DNA checkerboard.^116^, ^117^ The highest bacterial DNA yield was obtained from supragingival plaque, followed by tongue dorsum samples, while the mucosal swab sample yielded the lowest yield. Compositionally, the mucosal swab sample differed the most from the rest, while the two plaque samples (>80% similarity) and saliva and tongue sample (>90% similarity) formed two distinct subclusters. Interestingly, the red‐complex species (*Tannerella forsythia*, *P. gingivalis*, and *Treponema denticola*) increased with age, irrespective of the sample type.^116^ Cariogenic bacteria (*S. mutans*, *Streptococcus sobrinus*) were not limited to the dental surfaces and also increased with age.^117^ The lowest proportion of these microorganisms was found in subgingival plaque, while the mucosal swab sample contained the highest proportions of *S. sobrinus* and *S. mutans*.

Recently, findings that saliva and supragingival plaque in young children harbor very different microbial communities have been confirmed by 16S rDNA amplicon sequencing.^48^, ^118^ Plaque was shown to have a higher alpha diversity than saliva. A longitudinal study on maturation of the oral microbiome in 119 caries‐free children showed that both saliva and plaque undergo distinct compositional changes in the period from 1 to 4 years of age.^118^ At the level of the overall composition of the two sample types, plaque and saliva shared between 72% (1‐year‐olds) and 83% (4‐year‐olds) of the taxa. However, when individually paired plaque and saliva samples were compared, a large inter‐individual variation was observed, ranging between 0% and 65% in the proportion of shared taxa. In other words, a large proportion of taxa in supragingival plaque can be found in saliva, but this does not occur in every child. The factors influencing this variation still need to be determined.

Sampling young children, especially infants, is not always easy. Additionally, collecting samples in large cohort studies requires a vast amount of resources. Home‐sampling in a familiar setting by an instructed caretaker could provide an alternative. A recent study addressed the feasibility of home‐sampling by mothers in 2‐ to 15‐month‐old infants in comparison with sampling by a trained researcher (submitted). For this study, the mothers of 30 infants first received video instructions for collecting plaque, saliva, buccal, and tongue swab samples, after which they collected the samples, followed by repeated sampling by the researcher. Comparisons of the DNA yield/sample and the microbial composition showed that samples collected by mothers resembled those collected by the researcher, with the tongue sample being the most similar, and saliva the least.

##### Niche stability and comparability in adults

**FIGURE 3:**
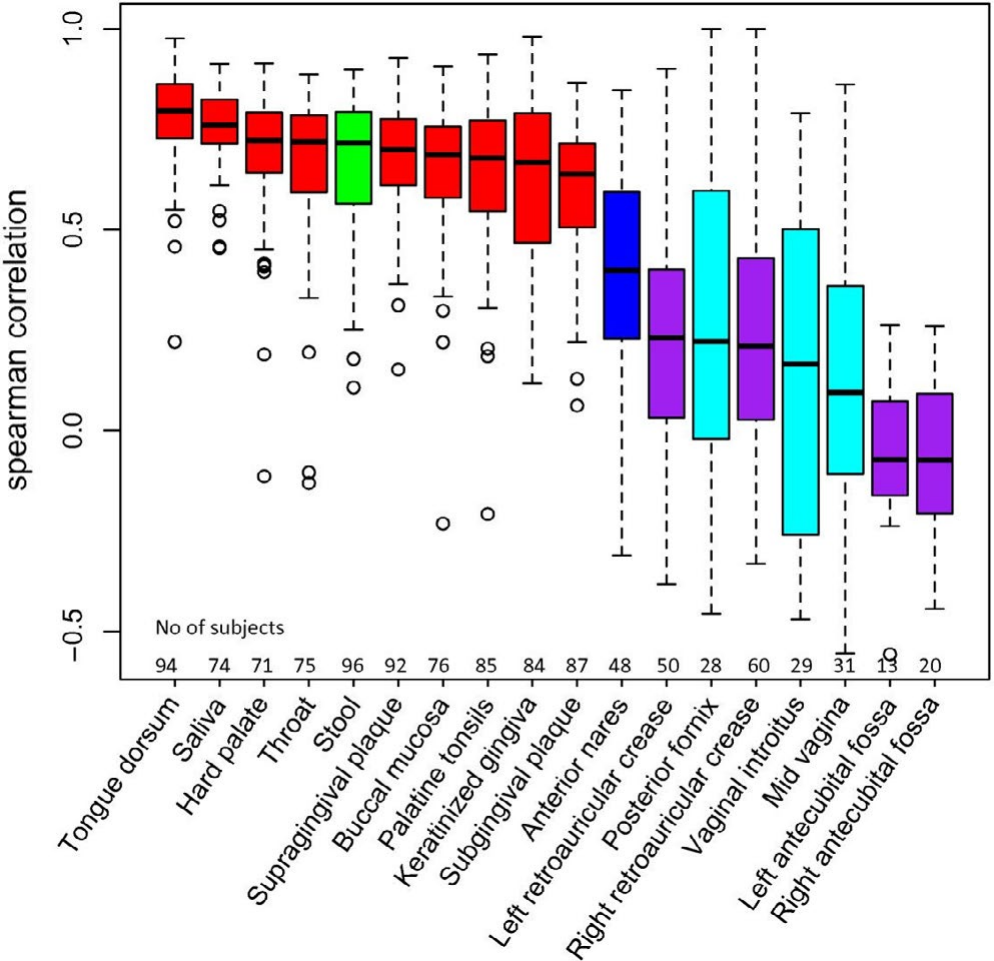
Microbial community stability over approximately 7 months. The higher the correlation coefficient, the more similar the temporal replicate samples collected within the Human Microbiome Project. In red, oro‐pharyngeal samples. Figure from^13^

As stated in the sections above, different intra‐oral niches will result in different microbiome outcomes. The choice of the sample could also be based on the robustness or temporal stability of the niche. Once established, oral microbial communities remain relatively stable, with tongue dorsum being the least variable in time, and subgingival plaque being the most (Figure 3).^13^ Intra‐individual temporal stability and inter‐individual differences were recently assessed in a study comparing tongue, saliva, and supragingival plaque in 10 individuals sampled at daily, weekly, and monthly intervals for up to 1 year.^119^ The authors found that plaque was significantly more variable than tongue or saliva. Additionally, they demonstrated that machine‐learning approaches could assign the samples to the right individual with 88%, 96%, and 97% accuracy, when using tongue, supragingival plaque, and salivary microbiome data, respectively. This suggests that tongue, although less variable, harbors a microbiome that is less discriminatory among adult individuals than saliva or plaque.

A relevant question regarding sample choice in periodontitis patients is if one has to collect subgingival plaque in order to assess the effects of interventions. Perhaps other samples such as saliva or tongue swab, both of which are less invasive and less time‐consuming, but known to differ significantly from dental plaque, are sufficient for observing differences over time or among study groups and could lead to conclusions comparable with those based on subgingival plaque samples. A recent study compared microbial profiles of 14 periodontitis patients before and after periodontal therapy, obtained from supragingival and subgingival plaque, chewing‐stimulated saliva, and tongue swab samples.^120^ The authors found that the relative abundances of 12 with periodontitis‐associated taxa, based on the red and orange complexes,^121^ were considerably higher in subgingival plaque than in any other niche. Nevertheless, the proportions of these taxa in subgingival plaque, saliva, and tongue correlated significantly. This was not the case for supragingival plaque, though. After periodontal therapy, the changes in the subgingival microbial composition (again, focusing on the same 12 selected taxa) were reflected in saliva, and correlated with the periodontal health status. These findings suggest that saliva could function as an alternative to subgingival plaque sampling in periodontitis patients.

Wei et al^122^ compared the microbial composition of subgingival plaque and buccal mucosa from healthy (N = 9) and periodontitis (N = 11 chronic, N = 12 aggressive periodontitis) patients. Their results discriminated between healthy and periodontitis patients based on the bacterial diversity of both buccal and subgingival microbiomes, with samples from healthy individuals having a lower bacterial diversity. Interestingly, microbial profile data ordination in principal coordinate analysis plots, as well as in individual operational taxonomic unit analyses, showed more distinct separation between healthy and periodontitis subjects based on their buccal samples than on their subgingival plaque. Larger studies should be performed to confirm these findings.

##### Dental calculus

Supragingival plaque data from healthy individuals (the Human Microbiome Project data set) were recently compared with data from modern and ancient dental calculus samples, where the modern calculus samples originated from both healthy subjects and periodontitis patients.^123^ There was a distinct separation in microbial profiles between plaque and calculus, with calculus samples having a higher proportion of periodontal disease‐associated species, irrespective of oral health status.

In summary, the choice of sample type(s) for a study on oral microbiome is not always straightforward and simple, and should be performed after evaluating relevance with regard to the study purpose and the feasibility in relation to the costs and logistics of the study.

### 3.7 Controls for microbiome studies

Another important aspect in the design of a microbiome study is planning and including both negative and positive controls to process alongside the biologic samples with each sample batch (Table 3).

**TABLE 3:** Negative and positive controls that should be included in microbiome studies

Negative controls	Positive controls
‐ Sampling blanks (eg, unused paperpoints, brushes, tubes with transport fluid) ‐ DNA extraction blanks ‐ Amplification blanks	‐ Known dilutions of known bacterial cells (mock community) or a standard sample as DNA extraction controls ‐ Known dilutions of known bacterial DNA (mock community) or a standard sample as amplification controls ‐ A standard sample or a mock community in studies with multiple sequencing runs to assess potential batch effects

#### 3.7.1 Negative controls

Inclusion of negative or blank controls allows assessing and correcting for potential contamination. Contamination with bacterial DNA from nonsample sources was never an issue in the studies targeting specific taxa or assessing microbial composition by culture. However, with an open‐end approach, as in amplicon sequencing of 16S rDNA, any bacterial DNA fragments can be amplified. For instance, the presence of bacterial DNA in selected batches of sterile paperpoints had a profound effect on the microbiome of peri‐implant sulcus samples.^124^ The samples collected from periodontally healthy sites without pockets presented with the lowest amount of bacterial DNA and were also the samples that had the highest proportion of the microbial sequences originating from DNA present in the paperpoints. This inverse relation between the sample template DNA and contaminant DNA has also been demonstrated on critically low‐biomass samples, such as placenta.^125^

In a recent review, a large number of genera included in the list of the common contaminant taxa belonged to a normal human (oral) microbiome, originating from the laboratory personnel.^126^ The sources of DNA contaminants range from the sampling materials and laboratory environment, researchers, and consumables, to DNA extraction kits and laboratory reagents.^126^ For example, infant nasopharyngeal samples clustered by the lot number of the DNA extraction kit used in the study.^127^ Besides the effects of the production lot, the DNA extraction blank controls across multiple studies have been shown to share several taxa,^127^ leading to the new term in this field, a “kitome”.^128^

Three types of negative controls should be included with each sample batch for microbiome analysis (Table 3): (a) the sampling blanks (eg, unused materials for sample collection such as paperpoints, swabs, brushes, and unused sample transport fluids) for assessment of contamination during the sample collection or transportation process; (b) the DNA extraction blanks for the assessment of contaminant DNA from the extraction kits; and (c) the no‐template amplification blanks for assessment of the contamination from the PCR reagents.

#### 3.7.2 Positive controls

In addition to the negative controls, two types of positive controls should be included and processed together with each batch of samples (Table 3). The first type is the DNA extraction positive control. For this, a mock community—known dilutions of known bacterial cells—or a standard sample (eg, a large volume of pooled saliva, aliquoted and stored at −80°C) are used. The second type is the DNA amplification positive control, which can be obtained by creating a DNA stock from either the mock community or from the standard sample. Positive controls allow assessment of experimental bias in sample processing. Additionally, in studies with more than one sequencing run, positive controls should be used to assess the variability among the different runs (batch effects).

## 4 SAMPLE PROCESSING

### 4.1 Sample collection methodology

Depending on the sample type, different sample collection methods are used; most of these were discussed in section 3.6 (Sample type choice). Irrespective of the sample type, the sampling method should have a low risk of introducing contamination to the sample. For instance, for subgingival plaque sampling, sterile curettes should be chosen over paperpoints if these cannot be claimed to be bacterial DNA‐free.^124^ Additionally, the sampling method should be feasible to perform under the conditions of the given study, considering the skills of the operator (eg, self‐sampling at home, sampling by a medical nurse or a trained dental professional).

### 4.2 Sample transport and storage

In the ideal situation, clinical samples are collected, placed in an empty, DNA‐free, sterile, and prelabeled tube, put on ice immediately, transported to the laboratory while on ice and stored at −80°C within 2 hours. Instant (snap) freezing, for example, by submerging the sample tube in liquid nitrogen is the best prerequisite to avoid changes in the sample, highly relevant for studies on microbial activity and not only taxonomic composition.

However, such ideal conditions are not always feasible, for example, as a result of sampling at home or in the clinic without a laboratory in the vicinity. Several studies have compared different sample preservation protocols for transportation and storage at an ambient temperature or in freezers at −20°C instead of −80°C (Table 4). There are expensive, commercially available products specifically aimed at bacterial DNA or RNA preservation, such as RNAProtect solution (Qiagen Inc.) that can be prefilled in the sample tubes, or previously mentioned specific sampling kits, such as OMNIgene saliva kit (DNA Genotek). To lower the study costs, alternative fluids, such as existing microbiologic sample transport media (eg, viable transport medium Gothenburg II, liquid dental transport medium) and antiseptic mouthwashes (eg, Scope), are used for sample preservation. Tris(hydrocymethyl)aminomethane‐ethylenediaminetetraacetic acid (TRIS‐EDTA) buffer combined with 0.5 M sodium hydroxide has been used for samples collected for DNA‐DNA checkerboard analyses.^129^, ^130^ The presence of ethylenediaminetetraacetic acid influences the magnesium concentration necessary for Taq polymerase activity,^131^ while the high pH will affect the optimum pH for the polymerase. Therefore, sample storage in TRIS‐EDTA buffer in 0.5 M sodium hydroxide is not a suitable approach for PCR‐based methods such as amplicon sequencing.

**TABLE 4:** Comparison of different oral sample transportation and storage methods

Preservation method	Microbial composition in comparison with other methods
OMNIgene saliva kit	Did not differ from samples without any additives if both directly stored at −80°C^113^ Storage for days at RT – no difference in alpha diversity from direct storage at −80°C^210^ Storage at RT for 14 d – different from storage directly at −80°C^210^ Reduced proportion of Firmicutes after storage at RT for 5 or 7 d compared with direct storage in LDTM medium at −80°C^210^
LDTM medium	Storage at RT for 2 d – no difference with direct storage at −20°C^210^
RNAProtect solution	Different composition after 2 wk at RT compared with direct storage in VMGII medium at −20°C^211^
VMGII medium	Storage for 2 wk at RT – no difference with direct storage in VMGII medium at −20°C^211^
Scope mouthwash	Storage at RT for 4 d – no difference with direct storage at −80°C^111^
TRIS‐EDTA/0.5 M NaOH (TE)	Storage at −20°C for 6 or 12 mo – different from direct processing (DNA‐DNA checkerboard) or storage for 6 wk at 4°C^130^ Storage for 12 mo – different from short‐term storage at different temperatures by DNA‐DNA checkerboard^129^

Abbreviations: EDTA, ethylenediaminetetraacetic acid; LDTM, liquid dental transport medium; TRIS, tris(hydrocymethyl)aminomethane; VMGII, viable transport medium Gothenburg II.

The studies above are small‐scale studies, highly heterogeneous in sample types, collection methods, storage conditions, and in approaches to assessing compositional differences. Additionally, it is not clear if, besides the sample stabilization, the additives influence microbial composition directly. This precludes a selection of the most optimal method for oral sample transportation and storage. For this, a systematic study comparing the available protocols still needs to be performed.

### 4.3 Bacterial DNA isolation

Only when the entire sample collection process is finished can the next step in sample processing—the isolation of bacterial DNA—start. It should be noted that it is advisable to wait with isolating DNA if it is not going to be processed further right away. DNA deteriorates and loses its quality during prolonged storage. Another reason for waiting with DNA isolation and processing all samples in one go is to reduce the risk of batch bias. Importantly, the samples belonging to different treatments should be randomized to avoid sample differences because of a batch effect, while time series (samples collected from the same individual at numerous time points) should preferably be processed together, in one batch, to reduce the inter‐sample variability.

Different sample types (eg, saliva, plaque, and mucosal tissue biopsy) will require different first steps in their processing. If the samples are stored in a transport fluid, this usually needs to be removed first. Thereafter, the obtained sample pellet should be subjected to cell lysis. This can be done either chemically (eg, with phenol/chloroform, TRIS‐EDTA buffer), enzymatically (eg, using lysozyme, proteinase K, achromopeptidase) or mechanically (eg, by bead beating in the presence of high‐density beads), but preferably by a combination of these methods. Cells of the Gram‐positive bacteria are generally more difficult to lyse and will require a mechanical lysis step.^132^

After cell lysis, the DNA needs to be separated from the lysate and purified. This is typically done using one of the commercially available DNA isolation kits developed for specific sample types and purposes (Table 5). In gut microbiome research, recent extensive study systematically compared 21 protocols for DNA isolation methods from fecal samples, and reported a large variation in DNA yield and quality between the protocols used.^133^ The protocols that performed best resulted in higher alpha diversity and included steps of mechanical lysis with zirconia beads and shaking.

**TABLE 5:** Studies of different DNA isolation methods from various samples of oral and nonoral origin

Sample type and reference	DNA extraction method/study design/samples per method/output	Lysis step	Main results
Fecal samples: Costea et al (2017)^133^	Part 1: compared variability introduced by 21 different DNA extraction methods and nonkit‐based protocols; N = 4 Illumina HiSeq WGS Part 2: three best performing protocols tested in four different labs. Next to this samples spiked with mock of 10 nonstool bacterial species; N = 6 Illumina HiSeq WGS	Chemical, enzymatic, and mechanical lysis by shaking or BB Mechanical lysis	Mechanical lysis, ZB, and shaking positively associated with diversity For both parts Qiagen QIAamp stool kit seems to perform best
Oral swabs, fecal, skin, marine, matrass samples: Marotz et al (2017)^212^	MoBio PowerMag Soil DNA isolation kit, adapted for three robotic systems vs column‐based method; sample size not reported Roche 454	Not mentioned	Magnetic method as good as column‐based method
Saliva spit, drool, and oral rinse samples: Lim et al (2017) ^113^	Part 1: spit directly stored at −80°C in OMNIgene or PBS. Three DNA isolation kits vs phenol chloroform; N = 20 Part 2: three saliva fractions, one DNA extraction method; N = 30 Illumina MiSeq	BB in lysis buffer followed by incubation at 70°C, then proteinase K	Part 1: Maxwell Kit performs best with spit samples in PBS. Part 2: different sample types lead to different DNA quality and quantity
Serially diluted saliva, upper airway communities: Biesbroek et al (2012)^134^	Four DNA extraction protocols; N = 32 Roche 454	Chemical/enzymatic lysis, BB, and BB/phenol protocols	At low DNA concentrations the microbial profiles deviated from the origin. DNA yield depends on isolation method. Results of BB/phenol with Agowa kit the best
Mock community, subgingival plaque: Abusleme et al (2014)^213^	Part 1: Mock community from 7 species: 4 lysis and 2 DNA isolation methods; N = 4 Part 2: Subgingival samples: 2 lysis and DNA isolation methods; N = 2 Roche 454	Crude chemical enzymatic lysis (C), C + DNeasy kit (Q), C and boiling + Q, BB + FastDNA Spin Kit (BB)	The method containing BB is the only one able to detect all species in the mock community
Saliva: Lazarevic et al (2013)^214^	Two lysis protocols; N = 3 Roche 454	Proteinase K with lysis buffer vs mechanical disruption in lysis buffer	Mechanical lysis results in higher abundance for certain species and a higher OTU richness
Oral mouthwash samples: Sohrabi et al (2016)^132^	Part 1: eight lysis methods; N = 4 Part 2: reproducibility of 3 consecutive DNA extractions; N = 3 DNA yield, DNA quality, qPCR on total bacteria, Firmicutes and human DNA	M1: lysozyme (L); M2: L + GB; M3: L + ZB; M4: Achromopeptidase (A), Tris‐EDTA & GB; M5: A, Tris‐EDTA and ZB; M6: L & A; M7: GB; M8: ZB	Cell lysis with ZB and L more effective than other methods
Saliva and dental plaque: Vesty et al (2017)^215^	Four DNA extraction methods; N = 3 DNA yield, Microbiome 16S V3V4 and Mycobiome ITS1. Illumina MiSeq	Mechanical, enzymatic, phenol/chloroform	Plaque: not affected by DNA isolation method. Saliva: three out of four DNA methods did not fully result in ITS (mycobiome) sequences
Oral mouthwash samples: Rosenbaum et al (2019)^216^	Eight DNA isolation protocols; N = 6 16S V6 and ITS1 region sequencing Illumina MiSeq	Methods including additional enzymatic and mechanical (BB) cell disruption steps	DNA yield differed between kits, microbiome results did not

Abbreviations: BB, bead beating; GB, glass beads; TRIS‐EDTA, tris(hydrocymethyl)aminomethane‐ethylenediaminetetraacetic acid; ZB, zirconia beads.

A similar study has not yet been performed on oral samples. Instead, there are numerous highly heterogeneous small‐scale studies, typically comparing a few DNA isolation protocols in different types of oral samples stored using different storage conditions, all introducing bias in the sample composition (Table 5). This makes advising regarding the best methodology difficult based on current literature. Therefore, studies like those conducted by Costea et al,^133^ but on oral samples, would be welcomed.

After DNA isolation, the DNA yield and quality have to be determined. For this, spectrophotometric DNA measurements by nanodrop (ThermoFisher Scientific) or agarose gel electrophoresis are frequently used. However, these methods are not particularly sensitive, and low biomass samples will result in unreliable measurements. To increase the sensitivity and accuracy, quantitative PCR on conserved regions of 16S rRNA gene can be used for bacterial DNA quantification.^134^

It is important for researchers performing more than one microbiome study and aiming to compare their findings with those from their own future studies that the same laboratory facilities and equipment are used, as well as the same sample processing protocols, while insuring that all procedures are performed under aseptic conditions.^126^

## 5 AMPLICON PREPARATION AND SEQUENCING

The steps after the sample DNA isolation, purification, and quantification are: (a) the amplification (preparation of individual amplicons or PCR products) of a specific part of the 16S rRNA gene of each sample together with a barcode that allows unique indexing of each amplicon; (b) preparation of the amplicon mix (pooling of individually barcoded amplicons into an equimolar mix); and (c) sequencing (reading the order of nucleotides) of individual sequences (reads) in this amplicon pool using one of the next generation sequencing technologies. There are some crucial decisions that should be made upfront.

### 5.1 Hypervariable region choice

The component of the small ribosomal subunit gene, the 16S rRNA gene, is approximately 1500 base pairs long and contains nine highly conserved parts, which are nearly identical in most bacteria, and nine hypervariable regions, parts of which have slowly evolved and can be used for discriminating different bacterial taxa. Unfortunately, different hypervariable regions evolved differently and there is no single region that would be able to distinguish all bacterial lineages.^135^, ^136^ The most optimal would be sequencing the entire gene, thus about 1500 bases. To date, this is possible with few sequencing technologies (Table 6). However, most studies currently use technologies that are high throughput and deliver shorter but high quality sequencing reads. For this, one should choose which region(s) or combinations of regions to target.

**TABLE 6:** An overview of next generation sequencing platforms and technologies

Company/technology	Platform	Maximum read length (bp)	Error rate	Maximum output	Estimated instrument cost	Estimated cost/billion base read	Maximum run time	Advantages	Drawbacks
Roche/Pyrosequencing	454 FLX+	700	0.1%	700 Mb	$100k	$10 000	23 h	Long read length	Expensive runs; homopolymer errors; phased out (2016)
Illumina Solexa/sequencing by synthesis	MiSeq	2 x 300	0.1%	15 Gb	$100k	$100	56 h	High accuracy	GC bias
	NextSeg 550	2 x 150	0.1%	120 Gb	$250k	$30	30 h	High accuracy; high throughput; low cost/base	GC bias
	HiSeq2500	2 x 125	0.1%	1 Tb	$750k	$30	6 d	High accuracy; high throughput; low cost/base	GC bias; short reads; expensive instrument
	NovaSeq6000	2 x 125	0.1%	6 Tb	$850k	$10	44 h	High accuracy; high throughput; low cost/base	GC bias; short reads; expensive instrument
ThermoFisher Scientific (Ion Torrent)/semi‐conductor sequencing	Proton	200	1%	10 Gb	$150k	$60	4 h	High accuracy; low cost/base; fast	Homopolymer errors
	PGM	400	1%	2 Gb	$50k	$400	7 h	High accuracy; low instrument costs; fast	Homopolymer errors; high cost per base
ThermoFisher Scientific (Life Technologies)/sequencing by ligation	SoliD 5500xl	2 x 50	0.01%	240 Gb	$595k	$40	10 d	High accuracy; High throughput; low cost/base	Very slow; short reads; expensive instrument
Pacific Biosciences/SMRT sequencing	RS II	15 000	15%	1 Gb	$700k	$400	4 h	Very long reads; fast; no amplification bias	High error rate^a^; Expensive instrument; high cost per base
	Sequel	30 000	15%	10 Gb	$350k	$85	20 h	Very long reads	High error rate
Oxford nanopore/nanopore sequencing	MiniON	>30 000	10%	20 Gb	$1k	$75	48 h	Very long reads; cheapest instrument; no amplification bias	High error rate
	PromethION	>30 000	10%	15 Tb	$160k	$10	48 h	Very long reads; lowest cost/base	High error rate

Abbreviations: bp, base pairs; GC, guanine and cytosine; PGM, personal genome machine; SMRT, single molecule, real‐time

^a^ High error rate without circular consensus sequencing.

The importance of the 16S rRNA gene hypervariable region choice was clearly illustrated in the early days of the next generation sequencing era using 454 pyrosequencing technology: results obtained from sequencing different hypervariable regions (V1‐V3, V4‐V6, V7‐V9) of subgingival plaque bacterial DNA differed significantly.^137^ For example, the genus *Fusobacterium* accounted for 18% of the sequences in the data set from V1‐V3, for 4% in the V4‐V6 data set, but was not detected at all in the data set from V7‐V9. Thus, the latter region was not discriminatory enough for this specific taxon, and the respective sequences were classified at a higher taxonomic level (eg, class, order, or phylum) instead.

To assist in the choice of region, especially if there is no previously published comparison available on the particular sample type targeted by different regions, there are tools available which allow in silico assessment of the taxa that could at least theoretically be distinguished by specific primers aimed to amplify specific hypervariable regions.^138^, ^139^

Since the introduction of the V4‐based Illumina MiSeq protocol,^140^ the V4 hypervariable region has been frequently chosen ahead of others. This is mainly because this region is entirely covered by the two 250 nucleotide paired‐end reads (thus, sequenced from both ends, creating a complete overlap), thereby reducing the error rate to a minimum.^141^

Besides the taxonomic differences introduced by the use of different hypervariable region(s), each primer pair will have their own primer bias: some taxa will be amplified more efficiently than others. Although most prokaryotes share the conserved regions of the 16S rRNA gene, there are no universal primers which will amplify all bacterial taxa. Some primer sets include degenerate bases (a mix of a number of possible bases instead of a single base) to reduce mismatches with bases of the 16S rRNA region and improve amplification of taxa that otherwise would not amplify or amplify less efficiently.

In summary, differences in universal primers and in hypervariable regions will affect the data obtained from the sequencing run. Again, as with sample processing, one should choose the methodology carefully and be consistent, as the results will not be directly comparable with studies using different methods.

### 5.2 Sequencing platform choice

Yet another design option is the choice of sequencing platform. The first sequencing method was developed in 1977 by Sanger et al^142^ and was revolutionary for that time, maintaining a monopoly until first of the next‐generation sequencing technologies, namely, pyrosequencing by 454 Life Sciences, became available in 2005. The advantage of next‐generation sequencing compared with traditional Sanger et al sequencing was the ability to sequence hundreds of thousands of sequences simultaneously (compared with a single sequence by Sanger et al sequencing) without the need of a cloning step before the sequencing step. The largest disadvantages were the short reads (only 50 base pairs in the earliest editions), high costs, and extremely high error rates. Since then this field has evolved at an immense speed.

Second generation sequencing (454, Illumina, Ion Torrent, SOLiD) has been followed by third generation (Pacific Biosciences of California, Oxford Nanopore Technologies). Currently, there are multiple platforms available to choose from, each using different technologies, with large variation in their costs, read length, and quality (Table 6). Third‐generation sequencing such as Pacific Biosciences single molecule, real‐time sequencing and nanopore sequencing do not require an amplification step before the sequencing and the signal is captured in real time.^143^ To date, however, these methods suffer from low accuracy and require either a production of consensus sequences (circular consensus sequencing PacBio) or application of extensive sequence correction tools.^144^

Currently, because of the high quality, broad availability, and relatively low sequencing costs, the Illumina MiSeq platform is most frequently chosen in published studies on the oral microbiome, followed by Illumina HiSeq2000. Comparison of the performance of these two platforms led to a conclusion that the results are consistent across the platforms.^145^ Sporadically, other platforms, for example in a study on saliva samples using the Ion Torrent PGM platform,^146^ or on saliva and dental plaque using Pacific Biosciences’ single molecule, real‐time platform,^147^ are used. Most likely, third‐generation sequencing platforms will gain in popularity in the future.

Because the cost of sequencing is no longer a limiting factor and next generation sequencing platforms have an extremely high throughput, the number of samples per sequencing run can be increased to support many more indices: 1536 or even 147 456 (384 × 384) unique combinations and thus number of samples per run.^141^, ^148^ Sequencing microbial communities such as human microbiome at a higher depth does not provide more information if sequenced at about 40 thousand reads per sample on MiSeq compared with about 1.2 million reads per sample on HiSeq platform.^145^ Increase in sequencing depth per sample (thus less samples with more reads per sample) would only be relevant if the “rare biosphere” is targeted.

## 6 BIOINFORMATICS

The sequences obtained using one of the next generation sequencing technologies need to be processed into a data set that can be used for testing the study hypothesis. In the early days of microbiome research, researchers had to rely on separate, custom‐made scripts, using a command line and requiring long computing times.^4^, ^149^ In the past decade, this field has evolved from numerous web‐interfaces and software packages that combine several tools to complete self‐contained data‐processing and analysis pipelines such as QIIME and mothur (Table 7). For the advantages and shortcomings of the majority of these tools, refer to systematic comparisons published elsewhere.^150^, ^151^

**TABLE 7:** An overview of 16S rRNA gene amplicon data‐processing software

Process	Tool	Description
Quality control	FastQC^217^	Quality control of raw sequencing data
Self‐contained analysis pipelines (including quality‐filtering, chimera removal, the construction of OTU tables, assignment of taxonomy, with or without data analyses)	QIIME^218^	Quantitative Insights Into Microbial Ecology. Software pipeline from raw sequencing data until data interpretation (visualization, statistical tests)
	QIIME 2^192^	Redesigned QIIME. Supports processing the sequence data as well as downstream analyses
	Mothur^219^	A single software package for the analysis of amplicon sequencing data
	MG‐RAST^220^	MetaGenome Rapid Annotation using Subsystem Technology, a web‐based pipeline, also used for the analysis of shotgun metagenomics data
	USEARCH (UPARSE)^221^, ^222^	Software that supports all steps necessary to produce an OTU table and some downstream analyses
	VSEARCH^223^	A free open‐source alternative to USEARCH that supports most but not all algorithms of USEARCH
Creation of single‐nucleotide resolution of sequences	Minimum Entropy Decomposition^163^	An information theory‐based clustering algorithm for sensitive partitioning of sequences. Provides single‐nucleotide resolution (oligotypes or MED nodes)
	UNOISE^162^, ^224^	An algorithm within USEARCH for error‐correction of the amplicons. Creates high resolution OTUs referred to as zOTUs
	DADA2^164^	Corrects Illumina‐sequenced amplicon errors, providing single‐nucleotide resolution as ASVs
	Deblur^165^	Produces sOTU with single‐nucleotide resolution (putative error‐free sequences); processes each sample independently

Abbreviations: ASVs, amplicon sequence variants; MED, minimum entropy decomposition; OTU, operational taxonomic unit; sOTU, sub‐operational taxonomic unit; zOTU, zero‐radius operational taxonomic unit

Below, we briefly summarize the data‐processing steps and issues that are of importance in generation of valid study outcomes.

### 6.1 Data quality‐filtering

First, the sequences have to be quality‐filtered: the bases or reads with low quality scores (assigned to each read during the sequencing run) have to be removed. There is no default way to filter low quality regions or reads. The filtering depends on the sequencing platform, pipeline, and specific filtering method used. Therefore, these details should be reported in manuscripts. Each read is assigned to its sample of origin based on the barcode or index sequence. If the barcode and the primer were part of the sequence, these are trimmed off. Paired‐end reads are merged. Reads not assigned to any samples, reads of insufficient length, or reads with ambiguous bases, are generally removed.^152^ Next, chimeras or sequences that result from chimeric amplification during the PCR process need to be identified and removed.^153^ One can choose a specific software for identification of chimeric sequences or rely on tools provided by the respective complete data‐processing pipeline.^150^ In QIIME the default method is ChimeraSlayer,^154^ while in mothur it is UCHIME.^155^

### 6.2 Sequence clustering in operational taxonomic units or single nucleotide resolution

#### 6.2.1 Operational taxonomic units

After quality‐filtering, sequences are usually grouped (clustered) in operational taxonomic units, typically at a 97% similarity level, which was proposed in the 1990s as an approximation of bacterial species.^156^ This threshold leads to a reduced contribution of potential errors introduced both by PCR and sequencing in the final data set, called an operational taxonomic unit table. There are three approaches for operational taxonomic unit clustering: a de novo approach (without external reference sequences),^157^ or reference‐based approaches which can be either closed (entirely reference‐based) or open. In closed reference‐based clustering, all sequences that do not match with a sequence in the reference database are discarded. The open reference‐based method is a combination of a closed‐reference method followed by de novo clustering of previously unmatched sequences. It has been shown that these different clustering approaches result in a different number and composition of operational taxonomic units.^158^, ^159^, ^160^ Also, a single operational taxonomic unit can contain groups of sequences that each could individually be assigned to a different, related taxon. Additionally, clustering the 16S rRNA gene fragment at a 97% similarity will underestimate bacterial diversity in the sample, especially in the oral microbiome, where several closely related taxa will be clustered in one operational taxonomic unit. A recent study on comparison of 97% threshold with clustering at higher similarity found that at a full length of the 16S rRNA gene 99% similarity was the most accurate in taxonomic assignment of the sequence, while for the V4 hypervariable region 100% similarity was the most optimal.^161^

#### 6.2.2 Single nucleotide resolution

To reduce the dependency from sequencing errors and to obtain a data set at a single nucleotide resolution, thus at 100% instead of 97% sequence similarity, different error‐correction or denoising approaches have become available (Table 7). Instead of operational taxonomic units, the data table will contain features at a 100% sequence identity or single nucleotide resolution, such as zero‐radius operational taxonomic units,^162^ oligotypes, or minimum entropy decomposition nodes,^163^ amplicon sequence variants,^164^ or sub‐operational taxonomic units.^165^ Sequences from soil samples, when processed into amplicon sequence variants, sub‐operational taxonomic units, zero‐radius operational taxonomic units, or operational taxonomic units at 97% similarity, resulted in slightly different final data tables and the results were similar at the community level but were not the same if alpha diversity was considered.^166^ This would become an issue if one aims to identify rare taxa from the background noise.

#### 6.2.3 Taxonomy assignment

Each feature (eg, operational taxonomic unit, zero‐radius operational taxonomic unit, or minimum entropy decomposition) in the data table needs to be assigned a taxonomy. This is done by comparing the sequences from the data set with the sequences in a 16S rRNA gene reference database. There are large databases, such as SILVA,^167^ Greengenes,^168^ and the Ribosomal Database Project,^169^ containing bacterial sequences from all areas of microbiology, and specific databases limited to a single bacterial habitat, such as HOMD^170^ and CORE,^171^both of which are limited to sequences of microbiota previously associated with the oral cavity. The advantage of using databases tailored for the oral microbes is their higher taxonomic resolution than the broad databases. On the other hand, oral samples, especially if originating from immunocompromised individuals or very young children, may contain sequences that are not normally found in the oral cavity but are common in other environments such as water or soil. Depending on the sample, a larger or smaller proportion of the sequences in the data set will not be assigned taxonomy using the oral database alone or will be classified at a very low resolution such as phylum or even domain level. Therefore, a taxonomy assignment with one of the broad‐range databases should be performed in parallel to the oral database.

### 6.3 Data analyses

#### 6.3.1 Assessment of study controls

One crucial step before addressing the research question and looking at the study outcomes is a critical assessment of the study controls (Table 3). Negative controls are contaminated if they present high DNA yield relative to the samples and a high number of sequencing reads per control, at or above the detection limit of the positive controls (if these were included at various dilutions). In such a case, the data from the samples which had low DNA yield, resulted in a low number of reads, or both, should be discarded. Next, the sequences dominating in the controls should be compared with those in the samples. After identification of the contaminants, these should be subtracted from the final data set and reported as such. One may use a very conservative approach by removing all taxa present in the controls, but this may lead to removal of taxa that are truly present in the samples. There are filtering approaches available that would avoid the aforementioned issue.^172^, ^173^, ^174^ Also, one can use predictive modeling provided by tools such as SourceTracker^175^ to identify putative contaminants in the data set.

In large‐scale studies involving several sequencing runs and processing batches, the data from the positive controls should be used to assess the run‐to‐run variability and a potential batch effect.

#### 6.3.2 Data normalization issue

Although sequencing is performed with an equimolar amplicon mix of the samples, there are always inaccuracies in library standardization and amplicon pool mixing and thus library size standardization, as well as in the sequencing process itself. These inaccuracies may lead to a 10‐ or even a 100‐fold range in the number of reads per individual sample, which in turn will influence the study results: the samples sequenced at a higher depth will have higher species richness (number of taxa) than those at a lower sequencing depth, without any biologic reason behind these differences. Therefore, the data need to be normalized before the downstream analyses can be performed.

Currently, the most commonly applied normalization is rarefaction of samples to an equal depth (random subsampling without replacement), which results in discarding the remaining reads as well as samples with a depth below the chosen threshold from the data set. Rarefying samples for library size normalization is present in all major data‐processing and analysis pipelines. This method is not ideal, as it may reduce statistical power because of the loss of information, especially if too many samples are removed as a result of not reaching the rarefaction threshold. Additionally, this method does not address the compositional nature of the data (see the next section). Rarefaction has received criticism when compared with other normalization methods based on statistical mixture models.^176^ However, other researchers have shown that random subsampling to an equal depth may actually outperform other methods^177^: for sample groups with large (10‐fold) differences in the mean sequencing depth, rarefying was shown to lower the false discovery rate compared with a normalization by distribution used in DESeq2.^178^ Another study addressing the normalization issue concluded that the best method will depend on the exact structure of the data.^179^ To date, there is no consensus on the best method for normalization, but one should be aware that the method used may impact the study results.^177^, ^179^

#### 6.3.3 Data compositionality issue

**FIGURE 4:**
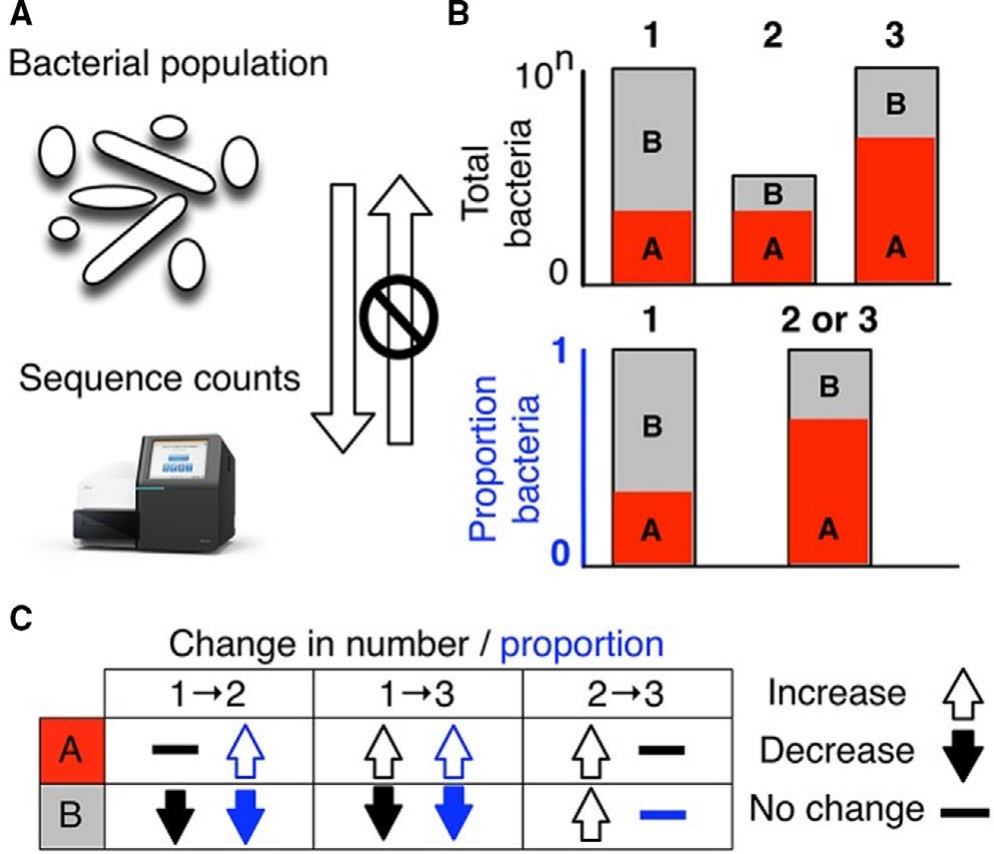
Illustration of the compositionality of the microbiome data, from Gloor et al^180^ (A) showing that the data obtained after sequencing cannot provide information on the absolute abundance of bacteria. The number of counts (reads) in the data set reflects the proportion of counts per feature (eg, operational taxonomic unit, gene) per sample, multiplied by the sequencing depth, thus the relative abundances. The bar charts in (B) show the difference between the bacterial count and the proportion of bacteria for two features, A (red) and B (gray) in three samples. Features A and B in samples 2 and 3 appear with the same relative abundance, although the absolute counts in the environment were different. The table in (C) shows real and perceived changes for each sample in transition from one sample to another

The compositionality of the data is an issue that is frequently underestimated or ignored in microbiome studies. Compositional data consist of relative abundances or proportions of various features (taxa), and the total (reads/sample) of it is arbitrarily imposed by the sequencing instrument.^180^, ^181^ Gloor et al^180^ clearly illustrated the importance of acknowledging that microbiome data should be treated as compositional (Figure 4). The compositional nature of microbiome data has large consequences on correlation analyses, as it is vulnerable to negative correlation bias and instability of correlation.^180^

To deal with compositional data, it is advised to perform logarithmic ratio transformation (eg, log‐ratio, centered log‐ratio, isometric log‐ratio, additive log‐ratio transformations).^180^ These transformations, however, suffer from the sparsity of the data (a high count of zeros in the data table; logarithm of zero is undefined). For that, specific methods can be applied such as implemented in the zCompositions R package^180^, ^182^ or pseudo‐counts can be used, although there is no consensus on the pseudo‐count value.^177^

In their work, Gloor et al^180^ provide a list of methods and downstream analyses that account for data compositionality. It is important to realize that ignoring the compositional nature of the data may lead to erroneous conclusions not based on true biologic differences.

#### 6.3.4 Downstream analysis tools

Finally, what remains is to make sense out of the data. Already in the planning stage of the study, one is advised to become acquainted with the amplitude of the downstream tools which can be used for analyzing the data, depending on the study design and hypothesis. For this, reading tutorials and user manuals of one of the major pipelines such as mothur or QIIME may be useful. These will include, but will not be limited to the alpha (within‐sample) and beta (between‐samples) diversity assessment, data visualization by ordination techniques such as principal component analysis or principal coordinate analysis, and the use of appropriate statistics. Some of these approaches have been clearly explained and illustrated by Goodrich et al.^19^

Beyond the tools implemented in the above‐mentioned data analysis pipelines, several software tools are available for comparison of two or more groups of microbial communities or for identifying differential taxa between the groups (Table 8).

**TABLE 8:** Examples of downstream analysis tools for 16S rRNA gene amplicon data

Tool	Description
MaAsLin^225^	*Multivariate Association with Linear Models* Finds associations between clinical metadata and microbial community abundance or functions
LEfSe^226^	*Linear discriminant analysis Effect Size* Is used for discovering “biomarkers” discriminating between groups
PICRUSt^227^	Predicts functional composition of microbial communities using 16S rDNA sequences and a database of reference genomes
Tax4Fun^228^	
STAMP^229^	*STatistical Analyses of Metagenomic Profiles* Tool for comparative metagenomics. This can be used on the results of PICRUSt or Tax4Fun. Provides statistical analyses and plots showing the different features (functions/taxa) between groups
Phyloseq^230^	Supports handling of microbiome data as well as analysis and visualization

Only recently, specific tools for longitudinal microbiome data sets have become available.^183^ The QIIME2 pipeline now supports analyses of time‐series data using q2‐longitudinal software plugin,^184^ while new dynamic models have been reconstructed from time series data.^185^

### 6.4 Reporting of the study, data deposition, and reuse

As already stated in the section on study metadata (section 3.5), each study should be reported in a way that study methods can be reproduced. A detailed description of the study population with the necessary metadata, detailed and properly referenced methods on sample collection procedure, sample processing, as well as the steps involved in data creation and processing, should be reported.

Recently, FAIR guiding principles for scientific data management and stewardship have been proposed.^186^ FAIR stands for Findable, Accessible, Interoperable, and Reusable, and refers to improved infrastructure that will support the reuse of scientific data. The majority of current research data are obtained with public funding and should therefore be publicly available.

#### 6.4.1 Data depository

Most journals but also research funding organizations require authors to make their sequencing data available. This could be “available upon request”, but most often data deposition is required in publicly available databases, such as the Sequence Read Archive (often referred to as the Short Read Archive) of the National Center for Biotechnology Information^187^ and the European Nucleotide Archive.^188^ Together with the data, a minimum amount of information on the experiment has to be provided, including details on sequencing, such as the target gene or gene region, the sequencing method used, and a reference to the publication with details regarding the study.^189^, ^190^ Both the Sequence Read Archive and European Nucleotide Archive deploy data standards and checks on data submission in collaboration with the Genomic Standards Consortium. However, it still remains the responsibility of authors to make the data and metadata publicly available.

#### 6.4.2 Data reuse

Usually an omics study, such as the microbiome, is published in a relatively condensed way, presenting the major findings of the study in the main and supplementary material. This, however, does not mean that the scientific value of the data is exhausted. For example, the data obtained in the aforementioned study on the effects of antibiotics ^42^ were reanalyzed by experts in data modeling and provided additional insights.^191^ Although the possibility of combining different microbiome data sets from several studies into a single data set and performing a meta‐analysis is currently still a challenge because of heterogeneity in study methodology, it will certainly become of high scientific value. This can only be possible if the data are findable (via an accession number in the publication) and well documented by all necessary metadata. Often, reanalysis of deposited data of a single study takes substantial effort and necessitates contacting the authors because of missing metadata, erroneous data accession numbers, or a lack of description in the processing steps, even in the accompanying publication.

In general, reproducing the study outcomes should always be possible if the methods used are provided in sufficient detail in the publication. To assist researchers, recent microbiome processing pipelines, for example, QIIME 2^192^ and DADA2/phyloseq,^193^ focus more on reproducible workflows.

## 7 OPEN QUESTIONS REGARDING THE QUALITY OF ORAL MICROBIOME STUDIES

Currently, the body of scientific knowledge is not always large or robust enough to pose a meaningful hypothesis for every microbiome‐based study.^194^ It is also likely that other confounding factors besides those listed in Table 1 influence the oral microbiome. This implies that both hypothesis‐driven studies as well as discovery‐based, exploratory studies should be performed. Both approaches are valuable, as long as the study aims and analysis methods are clearly determined beforehand. To ensure the robustness and applicability of the findings, the results need to be replicated, and this should be done using populations from geographically and culturally diverse populations. Studies that test and compare the validity and applicability of study power calculation methodologies are required, and user‐friendly versions with simplified instruction manuals of these tools are welcomed. In order to better understand the limitations and the effects of different choices on the outcomes, there is an acute need for a systematic comparison of the methods for oral sample storage and processing, for protocols for DNA isolation and primer choice for specific types of samples. Regarding study metadata acquisition, a questionnaire which could be used with all oral health‐related factors should be developed and validated for use in oral microbiome studies. Finally, within the scientific community, agreement on and adherence to minimum quality standards in the reporting and data deposition of (oral) microbiome studies is required.

To reach our ultimate aim—creation of knowledge that could be translated to clinical practice and personal oral care—we should take a lesson from the book *Rigor Mortis* by Richard Harris: “…to speed the development of medicine, biomedical science should actually slow down. This means taking on fewer projects and doing them more carefully. It means improving the quality of the scientific literature by publishing fewer, more careful papers.”^195^
